# The Nordic Maintenance Care Program: when do chiropractors recommend secondary and tertiary preventive care for low back pain?

**DOI:** 10.1186/1746-1340-17-1

**Published:** 2009-01-22

**Authors:** Iben Axén, Irene B Jensen, Andreas Eklund, Laszlo Halasz, Kristian Jørgensen, Fredrik Lange, Peter W Lövgren, Annika Rosenbaum, Charlotte Leboeuf-Yde

**Affiliations:** 1 The Karolinska Institute, Stockholm, Sweden; 2 Private practice, Stockholm, Sweden; 3 Private practice, Lund, Sweden; 4 Private practice, Linköping, Sweden; 5 Nordic Institute for Chiropractic and Clinical Biomechanics, Part of Clinical Locomotion Science, University of Southern Denmark, Denmark

## Abstract

**Background:**

Among chiropractors the use of long-term treatment is common, often referred to as "maintenance care". Although no generally accepted definition exists, the term has a self-explanatory meaning to chiropractic clinicians. In public health terms, maintenance care can be considered as both secondary and tertiary preventive care. The objective of this study was to explore what factors chiropractors consider before recommending maintenance care to patients with low back pain (LBP).

**Method:**

Structured focus group discussions with Swedish chiropractors were used to discuss pre-defined cases. A questionnaire was then designed on the basis of the information obtained. In the questionnaire, respondents were asked to grade the importance of several factors when considering recommending maintenance care to a patient. The grading was done on a straight line ranging from "Very important" to "Not at all important". All members of the Swedish Chiropractors' Association (SCA) were invited to participate in the discussions and in the questionnaire survey.

**Results:**

Thirty-six (22%) of SCA members participated in the group discussions and 129 (77%) returned the questionnaires. Ninety-eight percent of the questionnaire respondents claimed to believe that chiropractic care can prevent future relapses of back pain. According to the group discussions tertiary preventive care would be considered appropriate when a patient improves by 75% or more. According to the results of the questionnaire survey, two factors were considered as "very important" by more than 70% of the respondents in recommending secondary preventive care, namely frequency past year and frequency past 10 years of the low back pain problem. Eight other factors were considered "very important" by 50–69% of the respondents, namely duration (over the past year and of the present attack), treatment (effect and durability), lifestyle, work conditions, and psychosocial factors (including attitude).

**Conclusion:**

The vast majority of our respondents believe that chiropractic treatment can prevent relapses of back pain. When recommending secondary preventive care, past frequency of the problem is considered. For tertiary preventive care, the patient needs to improve considerably before a recommendation of maintenance care is made.

## Background

Low back pain (LBP) is a major public health problem in the industrialized world because of a high prevalence and subsequent high costs. As evidence suggests that the condition is recurrent in a large proportion of cases [1,2], it is important to learn more about secondary and tertiary prevention of LBP.

Chiropractors in Europe manage both acute and chronic LBP cases [3-7]. Although there is evidence of the efficacy of short-term manipulative treatment [8,9], a recent literature review [10] revealed no studies on the preventive effect of treating patients for periods of several months or even years. Nevertheless, most chiropractors seem to use some kind of long-term management approach [11,12], often referred to as maintenance care (MC). As implied by the name, this kind of treatment will attempt to maintain the level of improvement or function achieved by treatment [13], but also to minimize the risk of recurrence [14] or even to maintain optimal health [15]. Although MC is a well known management strategy among chiropractors [11,16] no evidence based definition exists [10] and, apart from a pilot study [17], its efficacy has never been scientifically tested. However, in order to do so, it would be necessary first to establish the criteria for its usage.

Work on defining and exploring the concept of MC has been initiated in the Nordic countries. In a questionnaire study from Sweden, chiropractors matched hypothetical cases with management strategies [18], and we conclude that there seems to be a general management culture to continue treating patients with LBP past the initial treatment period. We also conclude that the rather vague term of MC seems to have a self-explanatory meaning to chiropractors. In our opinion, chiropractors are often unaware of the different definitions of prevention commonly used by other health care professions. The term MC seems to cover both the concept of secondary and tertiary prevention [19]. In the case of secondary prevention, the aim of the treatment would be to prevent relapses in a patient with recurrent LBP. In a more chronic LBP case, tertiary preventive care would aim at maintaining the problem at an acceptable level.

The purpose of this study was to explore the decision-making process used by Swedish chiropractors when considering recommending MC for patients with LBP. Specifically, we wanted to identify 1) if the frequency of previous events of LBP is used in the clinical decision process for secondary prevention, and 2) if the level of improvement is taken into consideration in the case of tertiary prevention. In addition, we wanted to identify and investigate any other patient-related factors commonly taken into account when chiropractors consider recommending MC.

Our hypothesis was that a patient with no previous events of LBP would not be a candidate for secondary prevention, whereas one with many previous episodes would be. Also, we hypothesized that a certain level of improvement from chiropractic treatment was necessary before tertiary prevention would be considered.

## Method

### Developing the questionnaire

A group was formed which consisted of seven chiropractic clinicians with similar educational background and varying years of clinical experience (4–25 years) to work on clinic-based research projects together with an experienced researcher.

On the basis of a previous study [18], nine clinically relevant cases of LBP were defined by this team, some relating to secondary prevention (cases 5–9) and some to tertiary prevention (cases 1–4) (Additional File 1). All members of the Swedish Chiropractors' Association, SCA (In Swedish: Legitimerade Kiropraktorers Riksorganisation), were invited to participate in a workshop in conjunction with a biannual general assembly which took place in February 2007. The SCA consists of chiropractors with a Council for Chiropractic Education (CCE) or European Council of Chiropractic Education (ECCE) approved education. This approval ensures a sufficient academic standard from colleges in Europe and North America.

The workshop was designed as structured focus group discussions in order to collect qualitative data on clinicians' experiences and attitudes toward maintenance care. The participating chiropractors were divided into groups of 5; each with an assigned group moderator. The groups were asked to discuss the nine predefined cases and to make a decision whether they would recommend MC or not. These answers were written on flip-charts. The participants were also asked to note any additional factors that they considered important when reaching their clinical decision. After each case, a structured rotation of some group participants ensured the formation of new groups for the next case to be discussed.

Thirty-six of the 167 SCA members participated in the workshop. In Additional File 1, the definition of Maintenance Care used in the workshop, as well as the group responses can be seen. The participants agreed to a large extent on tertiary prevention (cases 1–5). If patients improved more than 75%, most groups would consider MC to be suitable. When discussing secondary prevention (cases 6–9), the participants agreed to recommend MC for patients with recurring LBP. However, all the groups mentioned other factors necessary to consider. These written qualitative responses were collected after the workshop to be carefully read and discussed by the research team. Words, expressions and themes were noted for each case, "weighed" according to the total number of times noted and, finally, categorized. The result of this process was a list of 14 factors concerning secondary prevention. This list subsequently guided the construction of a questionnaire. The questionnaire was tested on a small group (n = 8) of chiropractors for face validity and a few corrections were made. Several participants commented on the use of Likert type boxes for grading importance, so a straight line was chosen for sensitivity, in fact a Visual Analogue Scale (VAS). The final questionnaire, translated into English, is seen in Additional File 2.

### Design

A questionnaire was designed on the basis of the results from the workshop and distributed in a postal survey.

### Study participants

All 167 members of the SCA practising in Sweden were invited to participate in the questionnaire survey.

### Data collection

The questionnaire was mailed out in March 2007 together with a stamped return envelope and returned for analysis by June 2007. The questionnaire begins with the simple question:

"Do you believe that relapses of backache can be prevented with chiropractic treatment?" Those selecting the answers "yes, almost always" and "yes, sometimes" were asked to respond to a list of 14 factors possibly associated with secondary care. The chiropractors were asked to grade the importance of each factor on the VAS line, ranging from "Very important" to "Not at all important". One line was left blank as "other" should the chiropractor feel that there was something missing in the list.

### Ethical considerations

All participation in the workshop and questionnaire survey was voluntary. The respondents of the questionnaire could choose to register for future participation in research projects planned by the research group. In that case, they signed an attached informed consent form, allowing their name to be registered. No ethical permission is needed for such studies according to the Regional Ethics Committee regulations.

### Data analysis

The questionnaire analysis was done manually by IA and CLY. As the line used for grading importance was 80 mm long, our pre-hoc decision was that any mark below 20 mm (less than 25%) indicated that the item in question was of no importance to the chiropractor. The following 20 mm were considered to represent "a little important" factor, the next 20 mm a "moderately important" factor, and the last 20 mm (more than 75%) were considered to indicate that the item in question was "very important".

The numbers of "very important", "moderately important", "a little important" and "not important" answers for each factor were counted. In accordance with Bland and Altman [20], we quantified agreement pre-hoc. Thus, if any factor received a certain answer in 70% or more of the questionnaires, the decision was that the respondents had "good agreement". If the rate was between 50–69% it was considered "reasonable" and less than 50% was defined as "no agreement".

A test-retest was performed to test the reliability of the questionnaire. Twenty randomly selected chiropractors were sent the questionnaire a second time after 6 months. Their results were matched with their first recording. Using the four categories described above, a measure of agreement was chosen counting the number of total agreements between the first and the second recording, divided by the total number of observations. In addition, a second method was chosen: as a 20 mm change on the 100 mm VAS is considered to be a clinically important difference [21], we decided that any retest measures falling within 20% (i.e. 16 mm) of the corresponding original test measure was "agreement".

## Results

The questionnaire was completed by 129 (77%) of SCA members. The respondents who recorded their name (n = 92), were representative of SCA with regards to gender, age and years in clinical practise (Additional File 3).

The initial question of whether chiropractic care can be used preventively was by 126 participants answered with "yes, almost always" (n = 60) or "yes, sometimes" (n = 66). Thus, 98% of the responding chiropractors seem to support the concept of MC.

The hypothesis that *tertiary preventive care *is recommended to patients only if they improve considerably, was supported by the results obtained at the initial workshop (Additional File 1). According to the participating chiropractors, the patient should improve at least 50% before the clinician would recommend preventive care. When a patient shows a 76–80% improvement, most groups would consider MC to be suitable.

The hypothesis that *secondary preventive care *is recommended to patients with a history of previous LBP was also supported by the workshop (Additional File 1). However, the result from the workshop suggested that several other factors were taken into consideration before making this decision, factors further explored in the questionnaire survey. A summary of the replies to the specific questions obtained in the survey can be seen in Additional File 4. Two factors were found to have "good agreement" as "very important", namely i) the frequency of LBP over the past year, and ii) the frequency of LBP over the past 10 years. Eight factors out of 14 were found to have "reasonable agreement" as "very important", namely duration, (over the past year and of the present attack), treatment (effect and durability), lifestyle, work conditions, psychosocial factors and patient attitude. One factor had "reasonable agreement" as "not important", namely the patient's ability to pay for the treatment. Respondents failed to answer 8 times (0.4%) in 7 different questions.

An "other" factor was listed by 28 (22%) of the participating chiropractors. Most suggestions (n = 12) mentioned patient motivation (e.g. "patients' priorities", "if the patient wants a better health", "if the patient is expecting MC"). Some (n = 3) considered patient compliance (e.g. "patient's ability to follow advice"), some (n = 9) examination findings (e.g. "neurological status", "palpable dysfunction", "posture"), and a few (n = 4) miscellaneous answers were noted (e.g. "age", "body awareness", "effect on organic problems"). The research team would have placed the 12 patient motivation replies under "patient attitude". However, upon further scrutiny, 11 of the 12 respondents noting motivation as important had already noted "patient attitude" as "very important", so recoding this would not have affected the results.

Eighteen of 20 (80%) returned the retest questionnaire (Additional File 5), but only seventeen questionnaires were valid. Agreement was calculated in several different ways, and results ranged from 60% (perfect agreement by categories) to 72% (defining agreement as less than 16 mm using the line as a VAS, measuring continuous data), which we consider to be acceptable reliability.

## Discussion

### Developing the questionnaire

Focus group discussion is the dominant technique of collecting qualitative data [22]. This method offers a unique opportunity of gaining insights into experiences, opinions and perspectives otherwise less accessible, and can be used when constructing questionnaires.

Because the research topic was defined by experienced chiropractic clinicians, we chose a structured initial workshop, i.e. with a strong pre-existing agenda. This reduces the level of free conversation within the group but provides specific answers to the research questions. It also requires a high level of group moderator involvement, thus chiropractors with previous research experience were chosen and instructed on how to guide discussions and on how to extract the key words mentioned by several members of the group.

The participants in a group discussion determine the value of the data generated. Here, a fairly homogeneous group (same educational background and profession, all working in Sweden, most are acquainted) were asked to discuss experiences of clinical practise. The fact that the profession is small in Sweden (and so everybody knows each other), made the discussions flow easily. By changing the groups with each case, we hoped to avoid dominant personalities "taking over" and to allow for the more quiet participants also having their say. The participating chiropractors may not have been representative of the profession in Sweden though, as participation may have been attractive mainly for chiropractors living locally who had the opportunity to take the day off. Also, chiropractors feeling strongly about the topic may have chosen to attend, whereas those less interested may have abstained. As the participants were anonymous to the analysing research team, it is not possible to check for representativeness. This possible selection bias suggests caution when generalizing results.

The topic at hand, MC, did not seem to cause conflict; rather our impression was that the participants were eager to share and compare thoughts and experiences. The fact that there were majority decisions on most cases, suggests construct validity. This also suggests consensus on when to recommend MC among the participating chiropractors. Thus, the research effort seems appropriate. Further, the written responses captured further aspects of this domain useful in the construction of a future questionnaire, ensuring content validity.

At the initial workshop, the questions on when to recommend tertiary care were answered quite conclusively. When recommending prolonged care to a patient not expected to recover completely, the participating chiropractors agreed that the patient has to improve over 75%. This suggests that the chiropractors in this workshop agree on the concept of clinically relevant improvement. Previous work [23] has explored the use of long-term treatment in patients not improving, and concluded that therapists take the role of "health coach" in these instances, without focusing on the improvement per se. However, this topic was not specifically investigated in the present study.

### The questionnaire

In the initial workshop, the participating chiropractors supported the hypothesis that the past frequency of LBP determines the recommendation for secondary preventive care. However, all the groups stated that this clinical decision needs support from other factors as well. Interestingly though, according to the subsequent questionnaire survey, past frequency (the very factor expected to be important by the research group) was indeed chosen as "very important" by most respondents. This apparent contradiction may be due to the fact that the workshop presented clinicians with case scenarios and the questionnaire presented clinical factors. A clinical decision may require information about duration, frequency, pain intensity, work and social factors etc, which is why the workshop concluded that information about these factors was needed. However, when weighing the importance of these factors one by one in the questionnaire, the clinicians were indeed able to think in more general terms. So even if a number of additional factors are considered before recommending secondary preventive care, the most important one appears to be past frequency.

### Validity and reliability of the questionnaire

Rating the importance of each factor individually in this manner seems to be no problem, as the number of missing answers in the questionnaire was very small (0.4%). The initial workshop was attended by a small number of the SCA members who were anonymous to the analyzing group, which may result in bias. The questionnaire was, however, answered by a majority of the SCA members, most of who could be compared with the SCA member registry. The participating chiropractors were indeed found to be similar to the members of the SCA in terms of age, gender and years in practise. We therefore assume that the results from the questionnaire are likely to represent the Swedish chiropractors' opinions and experience.

As the area of MC is poorly investigated, no validated questionnaire exists to investigate chiropractors' opinions on when to use MC. However, because our questionnaire was constructed on the basis of discussions by focus groups of chiropractors, we feel that content validity exists. It was also tested by a small group of chiropractors before distribution for face validity and user friendliness. Third, the results confirmed our initial hypotheses, suggesting construct validity.

Testing reliability should always be in focus when constructing and testing a new questionnaire. However, the values of interest in our study (levels of importance) are not absolute; they are merely reflecting clinicians' attitudes. As such, precise consistency of this measure may be impossible to achieve. On the other hand, as they are reflective of clinical experience, they are thought to be relatively stable over time, thus we expect a respondent to answer within the same range in consecutive measurements. Using the four categories of importance renders "common" reliability tests (such as Intra Class Correlation) useless, as these require continuous data. Therefore, we chose a simple comparison of categories as agreement. Also, we used the analogy of our line to the VAS-line for defining an area of "no difference", i.e. agreement, and the results of the two reliability tests point in the same direction.

It is noteworthy that almost all (98%) of the study participants claimed to believe in the concept of MC. This figure strengthens our impression that even though not thoroughly described nor tested for its clinical validity, MC is widely accepted as a clinical strategy.

## Conclusion

The vast majority of Swedish chiropractors believe that chiropractic treatment can prevent relapses of LBP. The decision to recommend secondary preventive care to a patient with LBP is based on the past frequencies of the problem, in the past year and in the past 10 years. In addition, duration of the problem, treatment "effect", lifestyle, attitude, work conditions and psychosocial factors are considered. In the case of tertiary preventive care, the patient should improve at least 50% for a recommendation to be considered and if the improvement is over 75% the majority of study participants would recommend MC.

## Competing interests

The authors declare that they have no competing interests.

## Authors' contributions

IA was responsible for the design of the study, supervision of data collection, the analysis of data and the manuscript preparation. AE, LH, KJ, FL, PWL and AR were involved in the design, supervision of data collection and the analysis of data. CLY and IJ were supervising the study process and were involved in the analysis and manuscript preparation. All authors revised and approved the final manuscript.

## Supplementary Material

Additional file 1 **Nine hypothetical cases of low back pain presented for discussion in a workshop among Swedish chiropractors and the responses provided by 7 groups**. Click here for file

Additional file 2 **A questionnaire used to investigate the indications for maintenance care among 129 chiropractors working in Sweden.** Click here for file

Additional file 3 **The distribution of gender (%), age (%) and years in clinical practise (%) among identifiable respondents to a questionnaire survey among Swedish chiropractors as compared to members of the Swedish Chiropractors' Association, SCA.** Click here for file

Additional file 4 **Summary of replies to a questionnaire completed by 129 chiropractors working in Sweden, on factors considered important when recommending maintenance care.** Click here for file

Additional file 5 **Test-retest of a questionnaire measuring the importance of 14 factors considered by 17 chiropractors before recommending MC to a patient.** Click here for file
