# Supplementary material for: The Nordic Maintenance Care Program: when do chiropractors recommend secondary and tertiary preventive care for low back pain?
Source: Chiropr Osteopat. 2009 Jan 22;17:1. doi: 10.1186/1746-1340-17-1 (PMC2633010; doi:10.1186/1746-1340-17-1)
Supplement: Additional file 1 — Nine hypothetical cases of low back pain presented for discussion in a workshop among Swedish chiropractors and the responses provided by 7 groups. [file 1746-1340-17-1-S1.doc]

Nine hypothetical cases of low back pain presented for discussion in a workshop among Swedish chiropractors and the responses provided by 7 groups.

| CASE | QUESTION TO BE DISCUSSED:  Would you recommend MC to the patient IF.... | Yes | No | Yes  & No | No answer |
| --- | --- | --- | --- | --- | --- |
| 1 | .. he is 0-30% better (pain/ mobility/ quality of life) after 4 treatments? | 1 | 6 |  |  |
| 2 | ... he is 31-50% better (pain/ mobility/ quality of life)  after 4 treatments? | 1 | 5 |  | 1 |
| 3 | ... he is 51-75% better (pain/ mobility/ quality of life)  after 4 treatments? | 3 | 3 |  | 1 |
| 4 | ... he is 76-80% better (pain/ mobility/ quality of life)  after 4 treatments? | 5 | 1 | 1 |  |
| 5 | .. he previously has experienced LBP once a year? | 5 |  | 1 | 1 |
| 6 | ... he previously has experienced LBP four times a year? | 7 |  |  |  |
| 7 | ... he previously has experienced LBP once a month? | 7 |  |  |  |
| 8 | ...he previously has experienced LBP once a week? | 6 |  | 1 |  |
| 9 | ... he has never had LBP before, this was his first episode? | 3 | 3 | 1 |  |

LBP = Low back pain

| **MC = Maintenance care** = | **Treatment /follow up over a longer period of time after treating the initial problem.** |
| --- | --- |
| Secondary prevention = | You aim to prevent/prohibit recurrence of the initial problem. |
| Tertiary prevention = | You aim to keep the patient at a better/healthier level and prevent deterioration. |
| “Better” as used in tertiary prevention above = | Any outcome modality (e.g. pain, mobility, quality of life) chosen by the chiropractor as important. |
