# Supplementary material for: The Nordic Maintenance Care Program: when do chiropractors recommend secondary and tertiary preventive care for low back pain?
Source: Chiropr Osteopat. 2009 Jan 22;17:1. doi: 10.1186/1746-1340-17-1 (PMC2633010; doi:10.1186/1746-1340-17-1)
Supplement: Additional file 2 — A questionnaire used to investigate the indications for maintenance care among 129 chiropractors working in Sweden. [file 1746-1340-17-1-S2.doc]

A questionnaire used to investigate the indications for maintenance care among 129 chiropractors working in Sweden.

Hello all colleagues in Sweden,

As many of you know, a research team within the SCA is currently investigating Maintenance Care. A workshop was held at the half-yearly assembly in Stockholm to define WHEN the chiropractor sees it fit to recommend this type of treatment to a patient.

The analysis showed that to make this recommendation the clinician needs more information than what we supplied at the workshop. We have, on the basis of your suggestions designed this questionnaire. We hope that you have the time to help us by completing it.

The basis is this simple question:

**Do you believe that relapses of backache can be prevented with chiropractic treatment?**

(”chiropractic treatment” = all the treatment and advice that you give.)

Tick only ONE response:

No, never □ ⁭

Yes, sometimes □ ⁭

Yes, almost always □ ⁭

Don’t know □ ⁭

If your answer was” no, never” or”don’t know”: proceed to Question 2 on the next page.

If your answer was” yes, sometimes” or”yes, almost always” please answer Questions 1 and 2.

Question 1:

The case: A 40-year old man with low back pain. No other spinal pain or musculoskeletal pain. His X-rays are normal for his age. No “red flags” in the history or examination. He gets better (in regard to pain/mobility/quality of life) after a few treatments.

What factors would you consider if suggesting maintenance care to *prevent future low back problems*? Please mark on the line your assessment of the following:

**Very**  Not at all

important important

The patient’s level of pain at the first

consultation

The duration of the problem at the first

consultation

The triggering factors of his problem

The frequency over the past year

The frequency over the past 10 years

The total duration over the past year

The effectiveness of the treatment

**Very**  Not at all

important important

The “durability” of the treatment

Patient’s lifestyle (leisure, smoking, etc)

Patient’s working conditions

Patient’s psychosocial situation

Patient’s attitude

Patient’s ability to pay

Patient’s need to get back to work

Other. What ?………………………

Question 2:

We are going to need help in the future with data collection on a group of well defined patients who are treated chiropractically, some with and some without maintenance care.

Would you *possibly* be interested in helping out in such a research project?

If so, we need your name and telephone number for future contact:

Name…………………………………………………………………..

Phone (evening)……………………………………………………

Thank you for helping!

Iben Axén,

Annika Rosenbaum, Laszlo Halasz, Fredrik Lange, Andreas Eklund, Peter W. Lövgren

and Charlotte Leboeuf-Yde.
