# Supplementary material for: The Nordic Maintenance Care Program: when do chiropractors recommend secondary and tertiary preventive care for low back pain?
Source: Chiropr Osteopat. 2009 Jan 22;17:1. doi: 10.1186/1746-1340-17-1 (PMC2633010; doi:10.1186/1746-1340-17-1)
Supplement: Additional file 3 — The distribution of gender (%), age (%) and years in clinical practise (%) among identifiable respondents to a questionnaire survey among Swedish chiropractors as compared to members of the Swedish Chiropractors' Association, SCA. [file 1746-1340-17-1-S3.doc]

The distribution of gender (%), age (%) and years in clinical practise (%) among identifiable respondents to a questionnaire survey among Swedish chiropractors as compared to members of the Swedish Chiropractors’ Association, SCA.

|  | GENDER | | Age | | | | Years in practise | | | | | |
| --- | --- | --- | --- | --- | --- | --- | --- | --- | --- | --- | --- | --- |
|  | **M** | **F** | **< 30** | **31-40** | **41-50** | **51-65** | **0-4** | **5-9** | **10-14** | **15-20** | **21+** | **?** |
| SCA  n = 167 | 68 | 32 | 7 | 33 | 35 | 25 | 12 | 18 | 8 | 13 | 16 | 33 |
| Identifiable  study  population  n = 92 | 68 | 32 | 7 | 35 | 37 | 22 | 13 | 21 | 8 | 15 | 18 | 24 |
