# Supplementary material for: The Nordic Maintenance Care Program: when do chiropractors recommend secondary and tertiary preventive care for low back pain?
Source: Chiropr Osteopat. 2009 Jan 22;17:1. doi: 10.1186/1746-1340-17-1 (PMC2633010; doi:10.1186/1746-1340-17-1)
Supplement: Additional file 4 — Summary of replies to a questionnaire completed by 129 chiropractors working in Sweden, on factors considered important when recommending maintenance care. [file 1746-1340-17-1-S4.doc]

Summary of replies to a questionnaire completed by 129 chiropractors working in Sweden, on factors considered important when recommending maintenance care. 126 replies represent 100% as 3 respondents answered that they do not believe in MC, and thus were not asked to complete the whole questionnaire.

| Factor | N | Very  important (%) | Moderately important (%) | A little  important (%) | Not  important (%) |
| --- | --- | --- | --- | --- | --- |
| Level of pain | 125 | 10 | 15 | 27 | 47 |
| Duration present attack | 126 | 64* | 17 | 13 | 6 |
| Trigger factors | 125 | 44 | 29 | 15 | 12 |
| Frequency  past year | 126 | 79** | 12 | 7 | 2 |
| Frequency  past 10 years | 125 | 75** | 18 | 2 | 4 |
| Total duration  over past year | 126 | 67* | 19 | 10 | 5 |
| Effectiveness  of treatment | 126 | 63* | 26 | 9 | 2 |
| Durability of  treatment response | 123 | 55* | 28 | 12 | 2 |
| Lifestyle | 125 | 62* | 25 | 9 | 4 |
| Work conditions | 126 | 59* | 24 | 10 | 6 |
| Psychosocial  factors | 126 | 52* | 33 | 10 | 6 |
| Attitude | 126 | 61* | 23 | 7 | 9 |
| Able to pay | 125 | 6 | 18 | 24 | 52* |
| Return to work | 126 | 45 | 25 | 13 | 16 |
| Other | 28 | N = 25 | N = 1 | 0 | 0 |

** = good agreement (selected by >70% of respondents)

* = reasonable agreement (selected by 50-69% of respondents)
