# Supplementary material for: The Nordic Maintenance Care Program: when do chiropractors recommend secondary and tertiary preventive care for low back pain?
Source: Chiropr Osteopat. 2009 Jan 22;17:1. doi: 10.1186/1746-1340-17-1 (PMC2633010; doi:10.1186/1746-1340-17-1)
Supplement: Additional file 5 — Test-retest of a questionnaire measuring the importance of 14 factors considered by 17 chiropractors before recommending MC to a patient. [file 1746-1340-17-1-S5.doc]

Test-retest of a questionnaire measuring the importance of 14 factors considered by 17 chiropractors before recommending MC to a patient. Each box describes the chiropractors’ responses, the first number being the answer to the questionnaire in March 2007, the second the answer to the retest in September 2007.

18 chiropractors returned the questionnaire the second time, but one was not completed, leaving 17 valid responses.

| Factor | 1 | 2 | 3 | 4 | 5 | 6 | 7 | 8 | 9 | 10 | 11 | 12 | 13 | 14 |
| --- | --- | --- | --- | --- | --- | --- | --- | --- | --- | --- | --- | --- | --- | --- |
| Chiro-practor |
| 1 | 2-4 | 1-2 | 1-2 | 3-1 | 4-2 | 2-1 | 1-1 | 1-1 | 1-2 | 2-1 | 1-2 | 1-2 | 2-1 | 2-2 |
| 2 | 4-4 | 3-2 | 4-4 | 2-2 | 2-2 | 2-2 | 1-2 | 3-1 | 3-1 | 3-1 | 3-2 | 1-1 | 4-4 | 3-2 |
| 3 | 3-3 | 1-1 | 1-1 | 1-1 | 1-1 | 1-1 | 1-1 | 1-2 | 3-4 | 4-4 | 1-2 | 1-3 | 4-4 | 4-3 |
| 4 | 4-4 | 2-2 | 1-2 | 1-1 | 2-1 | 2-1 | 1-2 | 2-2 | 1-2 | 1-2 | 1-2 | 4-2 | 4-4 | 3-2 |
| 5 | 4-3 | 3-2 | 2-2 | 1-1 | 1-1 | 2-2 | 2-3 | 2-1 | 1-2 | 1-2 | 1-2 | 1-2 | 2-3 | 4-2 |
| 6 | 2-2 | 1-2 | 1-1 | 1-1 | 1-1 | 1-1 | 1-1 | 1-1 | 1-1 | 1-1 | 1-1 | 1-1 | 4-4 | 1-1 |
| 7 | 3-4 | 1-1 | 1-1 | 1-1 | 1-1 | 1-1 | 1-1 | 1-1 | 1-1 | 1-1 | 1-1 | 1-1 | 4-4 | 2-1 |
| 8 | 1-3 | 1-1 | 1-1 | 1-1 | 1-1 | 1-1 | 1-1 | 1-1 | 1-2 | 1-1 | 1-2 | 1-4 | 4-4 | 1-4 |
| 9 | 4-3 | 4-1 | 1-1 | 2-2 | 1-2 | 2-2 | 1-1 | 1-1 | 1-3 | 3-1 | 2-2 | 3-3 | 3-3 | 1-3 |
| 10 | 1-2 | 1-1 | 2-3 | 1-1 | 1-1 | 1-1 | 1-1 | 1-2 | 2-3 | 3-2 | 1-2 | 1-2 | 3-4 | 2-2 |
| 11 | 4-4 | 2-1 | 1-1 | 1-1 | 1-1 | 1-1 | 1-1 | 1-1 | 1-2 | 1-1 | 1-1 | 2-1 | 4-4 | 2-1 |
| 12 | 4-4 | 2-1 | 1-4 | 3-1 | 2-1 | 3-3 | 9-2 | 3-2 | 1-1 | 1-1 | 1-1 | 4-4 | 4-4 | 4-4 |
| 13 | 3-4 | 1-1 | 3-4 | 1-1 | 1-2 | 1-2 | 2-1 | 1-1 | 1-1 | 2-1 | 2-2 | 4-2 | 4-4 | 4-4 |
| 14 | 3-3 | 1-2 | 1-2 | 1-2 | 1-2 | 1-2 | 1-2 | 1-1 | 1-1 | 1-1 | 1-1 | 1-1 | 4-3 | 1-1 |
| 15 | 3-3 | 2-2 | 3-2 | 1-1 | 1-1 | 1-1 | 1-1 | 2-2 | 1-1 | 1-1 | 2-1 | 1-1 | 2-3 | 2-2 |
| 16 | 4-4 | 1-3 | 1-1 | 1-1 | 1-1 | 1-1 | 1-1 | 1-1 | 1-1 | 1-1 | 1-1 | 1-1 | 4-4 | 1-1 |
| 17 | 3-4 | 2-1 | 3-1 | 1-1 | 2-2 | 3-3 | 1-1 | 1-1 | 3-3 | 1-1 | 4-2 | 1-1 | 3-3 | 1-1 |
|  |  |  |  |  |  |  |  |  |  |  |  |  |  |  |
| Total agreement = 142 replies, 1 step difference = 73 replies, > 1 step difference = 23 replies Total: 238 replies | | | | | | | | | | | | | | |
